# Programmed cell death 1 pathway inhibitors improve the overall survival of small cell lung cancer patients with brain metastases

**DOI:** 10.1007/s00432-022-04121-y

**Published:** 2022-06-23

**Authors:** JiaYu Chang, XuQuan Jing, Ying Hua, KaiXing Geng, RuYue Li, ShuangQing Lu, Hui Zhu, Yan Zhang

**Affiliations:** 1 Department of Oncology, Shandong Cancer Hospital and Institute Affiliated to Shandong First Medical University & Shandong Academy of Medical Sciences, Jinan, 250117 China; 2 Tianjin Medical University, Tianjin, 300060 China; 3 Zaozhuang Tumor Hospital, Zaozhuang, 277102 China; 4 Weifang Medical University, Weifang, 261053 China

**Keywords:** Programmed cell death 1 (PD-1), Programmed cell death ligand 1 (PD-L1), Small cell lung cancer, Brain metastases, Immune checkpoint inhibitors (ICIs)

## Abstract

**Purpose:**

The objective of this study was to evaluate the safety and efficacy of immune checkpoint inhibitors in small cell lung cancer patients with brain metastases.

**Methods:**

We retrospectively reviewed the records of small cell lung cancer patients with brain metastases treated with chemotherapy and radiotherapy for brain metastases with or without immune checkpoint inhibitors at our institution from January 2019 to January 2021. Patients were divided into two groups. In Group A, patients received chemotherapy and radiotherapy for brain metastases. In Group B, patients received chemotherapy, radiotherapy for brain metastases and at least four cycles of immunotherapy. Overall survival and intracranial progression-free survival were assessed using Kaplan–Meier estimates and Cox regression models.

**Results:**

A total of 282 patients were enrolled in our study. At the end of the study (May 12, 2021), the median overall survival was 13.3 months among 218 patients in Group A and 33.4 months among 64 patients in Group B (hazards ratio [HR] 0.320, 95% confidence interval [CI], 0.189–0.545, *P* < 0.001). Both univariate and multivariate analyses suggested that two factors were significantly correlated with overall survival: the inclusion of immunotherapy in the regimen and the presence of extracranial metastases. The median intracranial progression-free survival was 6.93 months in Group A and 10.73 months in Group B (HR = 0.540, 95% CI, 0.346–0.841, *P* = 0.006). The intracranial objective response rate of Group B was greater than that of Group A, but the intracranial disease control rate was similar between the groups.

**Conclusion:**

Immunotherapy plus chemotherapy and radiotherapy for brain metastases showed promising efficacy for small cell lung cancer patients with brain metastases.

## Introduction

Small cell lung cancer (SCLC) is an exceptionally aggressive subtype of lung cancer, and over 20% of SCLC patients present with brain metastases (BMs) at the time of initial diagnosis (Cagney et al. 2017; Lamba et al. 2021). In addition, 50% of SCLC patients will develop BMs at some point during the course of the disease (Takahashi et al. 2017). These patients have limited therapeutic options. Most patients have a poor prognosis with a 5-year survival rate less than 5% (Li et al. 2021). Chemotherapy has limited efficacy due to the blood–brain barrier, blood-cerebrospinal fluid barrier, high interstitial fluid pressures and abnormal local perfusion (Askoxylakis et al. 2017). A significant number of patients respond to radiotherapy, but the response duration and survival were not ideal. The median overall survival (OS) was 6.5 months among SCLC patients who received stereotactic radiosurgery (SRS) and only approximately 5 months among those who received whole-brain radiation therapy (WBRT) (Bernhardt et al. 2018; Rusthoven et al. 2020).

Neurosurgical resection helps decrease the intracranial pressure caused by BMs (1–3 BMs) and offers prolonged survival when followed by adjuvant radiotherapy (Mahajan et al. 2017; Patchell et al. 1998).

Two randomized clinical trials, IMPOWER133 (Liu et al. 2021) and CASPIAN (Goldman et al. 2021), showed that programmed cell death 1 ligand 1 (PD-L1) inhibitors (atezolizumab or durvalumab) combined with platinum-based doublet chemotherapy improved OS (overall survival) versus chemotherapy in extensive-stage small cell lung cancer (ES-SCLC) patients. Subgroup analysis of the CASPIAN clinical trial suggested that SCLC patients with BMs had OS benefit from durvalumab. Programmed cell death protein 1 (PD-1) inhibitors also showed activity against BMs from non-small-cell lung cancer (NSCLC) with PD-L1 ≥ 50% (Metro et al. 2020). However, in IMPOWER133, the OS benefit for BM patients was not observed. Therefore, it remains unclear whether SCLC patients with BMs gain OS benefit from PD-1/PD-L1 inhibitor treatment. The objective of our study was to determine the efficacy of additional immune checkpoint inhibitors (ICIs) in the treatment of SCLC patients with BMs.

## Materials and methods

### Patient selection

We retrospectively reviewed the electronic medical records of SCLC patients with BMs in our hospital between January 2019 and January 2021 with the last follow-up date of May 12, 2021. Patients who matched the inclusion criteria were enrolled in the study. Inclusion criteria included (1) age > 18 years old; (2) Eastern Cooperative Oncology Group performance status (ECOG PS) of 0–2; (3) pathologically confirmed SCLC; (4) contrast-enhanced computed tomography (CT) or gadolinium-enhanced magnetic resonance imaging (MRI) confirmed BMs; (5) received at least four cycles of chemotherapy and radiotherapy for BMs with or without no less than four cycles of immunotherapy, and the treatment line when ICIs were applied was not restricted; and (6) complete medical records were available. The exclusion criteria were as follows: (1) patients who received less than four cycles of chemotherapy or who did not complete the radiation treatment and (2) incomplete medical records. According to the different treatments, patients were divided into two groups. In Group A, patients received chemotherapy and radiotherapy for BMs (CRT). In Group B, patients received CRT and immunotherapy (≥ 4 cycles).

The objective response was evaluated according to RECIST version 1.1. The death date and cause of death were followed up by telephone.

### Endpoints

Primary endpoints: Overall survival (OS) was defined as the time from BM diagnosis to death from any cause or last follow-up.

Secondary endpoints: Intracranial progression-free survival (IPFS) was defined as the time from radiotherapy for BMs to the date of intracranial objective disease progression or death from any cause in the absence of progression. Notably, when calculating the IPFS time in Group B, patients who received immunotherapy before, during or after radiotherapy within 1 month were enrolled, but those who received immunotherapy after more than 1 month were excluded. The intracranial objective response rate (IORR) was defined as the proportion of patients with a complete or partial intracranial response at least one visit. Intracranial disease control rate (IDCR) was defined as the proportion of patients with a complete or partial intracranial response or stable disease on at least one visit. Distant brain failure (DBF) time was defined as the interval between the end of radiotherapy for BM and the first occurrence of new intracranial lesions.

IPFS and objective response were assessed according to RANO criteria.

Intracranial and extracranial lesions were evaluated after every two cycles of treatment. BMs were assessed by MRI or CT and extracranial lesions were evaluated by CT. We tried to use the same imaging examination methods to evaluate the efficacy. Considering that different imaging methods may affect the interpretation of the outcomes, we tried to use the same examination method to assess the results before and after treatment.

### Statistical analysis

We used SPSS 23.0 software for statistical analysis. Categorical data were compared with Fisher’s exact or chi-squared tests. The Kaplan–Meier method was used to estimate the rates of OS and IPFS. To estimate OS differences among groups, the log-rank test was used. We used Cox regression models for multivariate analysis. All *p* values were based on the score test for a two-sided hypothesis. Two-sided tests with *p* < 0.05 were considered statistically significant.

## Results

### Patient characteristics

Two patients in Group B were excluded when data were analyzed because four cycles of systematic treatment were not completed. In one patient, treatment was incomplete due to grade 3 myocarditis only after one cycle of ICIs plus chemotherapy, and the other patient suffered from grade 3 pneumonia after two cycles of systematic treatment. Finally, a total of 282 SCLC patients with BMs were admitted to our study, including 218 patients in Group A and 64 patients in Group B. The median follow-up time was 18.5 (4.13–37.9) months in Group A and 12.3 (5.10–28.07) months in Group B. The median age of the patients at the time of diagnosis of BM was 61 years (ranging from 31 to 81 years). The median size of brain metastases was 16.31 mm (2–58 mm). Between the two groups, baseline patient characteristics (Table 1) and treatment (Table 2) were not significantly different. BM resection was rarely performed. Approximately seven types of ICIs were applied in Group B (Table 3). Among the 64 patients, 6 received 2 types of drugs, including 4 who received PD-1 and PD-L1 monoclonal antibodies and an additional 2 who used two different PD-1 monoclonal antibodies during treatment. No one received prior SRS. The SRS was delivered with a total dose of approximately 37.5–60 Gy by 8–20 fractions (2–6 Gy each time), and WBRT was typically performed with a total dose of 30–50 Gy by 10–25 fractions (2–3.5 Gy every time). Among the 40 patients who received PCI before BM occurred, 24 were treated with SRS after BM occurred, 9 received WBRT and 7 received WBRT-boost or SIB-IMRT. Among the 242 patients without prior PCI before BM occurred, 25 were treated with SRS after BM occurred, 121 accepted WBRT and 96 received WBRT-boost or SIB-IMRT. After documenting intracranial progression, 16 patients accepted best supportive care, 2 patients received ventriculo-peritoneal shunts to reduce intracranial pressure, and 79 patients received anticancer treatment. Of the 79 patients, 55 patients accepted systematic treatment (chemotherapy, anlotinib with or without immunotherapy), 14 patients received SRS plus systematic treatment, and the other 10 individuals only received radiotherapy for BMs (4 WBRT, 5 SRS and 1 SIB-IMRT).

**Table 1:** Baseline patient characteristics

Variable	Group A *N* = 218(%)	Group B *N* = 64 (%)	*P* value
Median age, years			0.087
≤ 60/ > 60	98(45.0)/120(55.0)	37(57.8)/27(42.2)	
Gender			0.457
Male/Female	173(79.4)/15(20.6)	48(75.0)/16(25.0)	
ECOG status			0.798
0–1/2	206(94.5)/12(5.5)	61(95.3)/3(4.7)	
Disease stage at diagnose			0.841
Limited stage/extensive stage	84(38.9)/132(61.1)	24(37.5)/40(62.5)	
Number of BM			0.814
1–3/4–10/ > 10	119(55.3)/66(30.7) /30(14.0)	34(53.1)/19(29.7)/11(17.2)	
Diagnosis of BM and LC			0.211
Subsequently/Synchronous	151(69.3)/67(30.7)	39(60.9)/25(39.1)	
Smoking			0.430
Yes/No	131(60.1)/87(39.9)	35(54.7)/29(45.3)	
ECM			0.222
Yes/ No	114(52.3)/104(47.7)	39(60.9)/25(39.1)	
Symptomatic BM			0.501
Yes /No	71(32.6)/147(67.4)	18(28.1)/46(71.9)	

*Statistically significant, *ECOG* Eastern Cooperative Oncology Group, *BM* brain metastases, *LC* lung cancer, *ECM* extracranial metastases

**Table 2:** Treatment

Variable	Group A *N* = 218(%)	Group B *N* = 64 (%)	*P* value
PCI			0.652
Yes/No	30(13.8)/188(86.2)	10(15.6)/54(84.4)	
Radiation for LC			0.739
Yes/No	149(71.3)/60(28.7)	47(73.4)/17(26.6)	
Resection of BM			0.658
Yes/No	2(0.9)/216(99.1)	1(1.6)/63(98.4)	
Radiation modality for BMs			0.486
WBRT/WBRT-boost or SIB-IMRT/SRS	96(45.3)/82(39.7)/34(16.0)	28(44.4)/21(33.3)/14(22.2)	
Follow-up imaging technique			0.198
CT/MRI	26(13.3)/169(86.7)	11(20.4)/43(79.6)	

*Statistically significant, *PCI* prophylactic cranial irradiation, *LC* lung cancer, *BM* brain metastases, *WBRT* whole-brain radiation therapy, *WBRT-boost* whole brain radiotherapy with consecutive boost, *SIB-IMRT* integrated simultaneous integrated boost intensity-modulated radiotherapy, *SRS* stereotactic radiosurgery, *CT* computed tomography, *MRI* magnetic resonance imaging

**Table 3:** Immune checkpoint inhibitors applied during radiotherapy for brain metastases in group B

Drug name	Number	Dose	Frequency (weeks)
Durvalumab	14	10 mg/kg	2
		1000 mg	3
		1500 mg	4
Atezolizumab	14	1200 mg	3
Nivolumab	6	200 mg	2
Toripalimab	6	240 mg	3
Tislelizumab	8	200 mg	3
Sintilimab	18	200 mg	3–4
Camrelizumab	4	200 mg	3

### Survival prognosis and clinical efficacy

#### OS analysis

Univariate and multivariate analyses suggested that two factors were related to the OS of ES-SCLC patients with controlled BMs after radiotherapy. A higher extracranial metastasis rate was correlated with worse OS, whereas immunotherapy was an independent prognostic factor associated with improved OS.

**Fig. 1:**
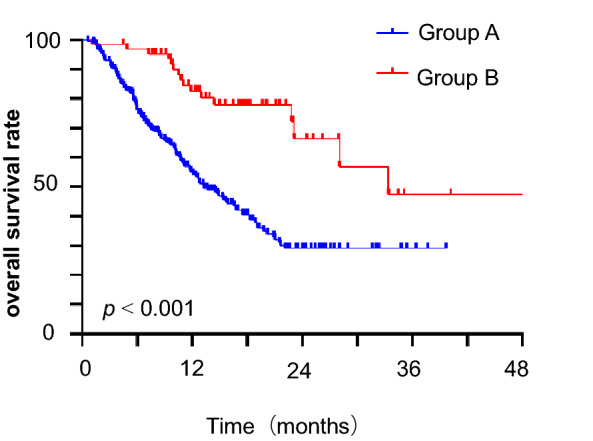
Kaplan–Meier graph of overall survival for Groups A and B

At the time of data analysis, at least four cycles of immunotherapy plus CRT were associated with a significant improvement in OS versus the CRT group (hazard ratio [HR] 0.320, 95% confidence interval [CI], 0.189–0.545, *P* < 0.001). A total of 127 patients in Group A died, and approximately 41.5% of patients in Group A were still alive. Sixteen of 64 patients in Group B died, and approximately 75% of the patients were still alive. The median OS was 13.3 months for Group A and 33.4 months for Group B. The 1-year OS rate was 82.6% in Group B and 54.1% in Group A (Fig. 1). Among the 127 patients in Group A, the reason for death was unknown, and 41 of these patients died of central nervous system (CNS) progression. Finally, five patients in Group B experienced CNS-related death. Fewer people died from uncontrolled CNS disease in Group B (*P* = 0.040).

**Fig. 2:**
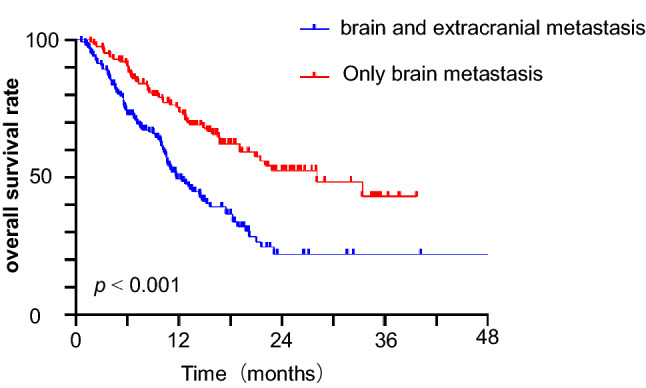
Kaplan–Meier graph of overall survival of SCLC patients with BMs and ECM versus those with only BMs

Extracranial metastasis (ECM) status was associated with worse OS. There were 128 patients with only BMs in our study, whereas 153 patients had both BMs and ECM. Until May 2021, more patients survived in the BM only group compared with the BMs and ECM groups (60.2 versus 39.9%). The median OS time of SCLC patients with only BMs was 28.1 months compared with 11.8 months for patients complicated with BMs and ECM (HR 0.429, 95% CI 0.285–0.646, *P* < 0.001). The 1-year OS rate was 72.8 versus 49.3% (Fig. 2).

The results of univariate and multivariate analyses of factors affecting OS are presented in Table 4.

**Table 4:** Univariate and multivariate analysis of overall survival

Variable	Univariate				Multivariate	
	HR	95% CI	*P*	HR	95% CI	*P*
Age			0.598			0.790
< 61/ ≥ 61	1.093	0.786–1.520		0.953	0.670–1.357	
Gender			0.145			0.589
Male/female	0.730	0.477–1.117		0.856	0.487–1.505	
ECOG status			0.544			0.499
0–1/2	0.801	0.391–1.640		0.759	0.341–1.689	
Number of BMs						
1–3 4–10 > 10	1.656 1.899	1.146–2.394 1.195–3.017	0.004* 0.007 0.007	1.442 10,812	0.925–2.248 0.954–3.439	0.141 0.106 0.069
Disease stage at diagnosis			0.565			0.534
Limited/ extensive	1.106	0.786–1.556		0.859	0.531–1.388	
Diagnosis of BM and LC			0.085			0.004*
Subsequently/synchronous	0.731	0.511–1.045		0.503	0.315–0.804	
PCI			0.377			0.639
No/Yes	0.805	0.496–1.305		0.857	0.449–1.635	
Smoking status			0.170			0.265
No/Yes	1.266	0.903–1.775		1.288	0.825–2.010	
Symptomatic BM			0.676			0.657
No/Yes	1.078	0.759–1.531		1.098	0.727–1.659	
Radiation for LC			0.114			0.374
No/Yes	0.747	0.520–1.074		0.820	0.530–1.270	
ECM			< 0.001*			< 0.001*
Yes /No	0.455	0.321–0.643		0.429	0.285–0.646	
Group			< 0.001*			0.005*
Group A/Group B	0.314	0.187–0.529		0.468	0.277–0.792	

*Statistically significant, *HR* hazard ratio, *CI* confidence interval, *ECOG* Eastern Cooperative Oncology Group, *BM* brain metastases, *LC* lung cancer, *PCI* prophylactic cranial irradiation, *ECM* extracranial metastases

Among patients who were complicated with only BMs, multivariate analysis showed that two factors were significantly correlated with OS. Patients who received additional immunotherapy had longer OS than those who did not (HR 0.206, 95% CI 0.65–0.651, *P* = 0.007). A greater number of BMs was related to worse OS (*P* = 0.015).

Among patients with BMs and ECM, additional immunotherapy was associated with superior OS (HR 0.371, 95% CI 0.207–0.663, *P* = 0.001). Synchronous BMs were associated with longer OS (HR 0.436, 95% CI 0.256–0.743, *P* = 0.002).

#### Cox regression analysis for IPFS

**Fig. 3:**
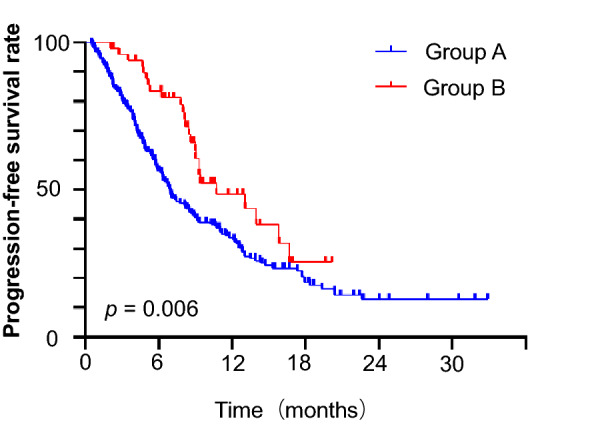
Kaplan–Meier graph of IPFS for Groups A and B

Our study found that additional immunotherapy prolonged the IPFS of SCLC patients with BMs. The median IPFS was 6.93 months for Group A and 10.73 months for Group B (HR 0.540, 95% CI 0.346–0.841, P = 0.006). IPFS at 12 months was 33.0 versus 48.5%, and IPFS at 18 months was 18.5 versus 25.4% (Fig. 3). A total of 68 patients in Group A and 7 in Group B died before the occurrence of intracranial disease progression.

**Fig. 4:**
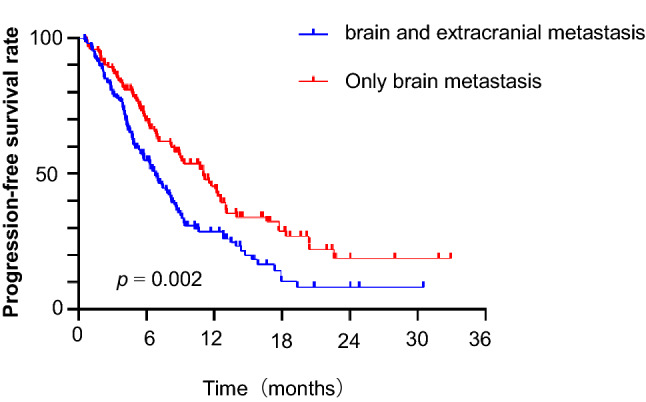
Kaplan–Meier graph of IPFS for SCLC patients with BMs and ECM versus those with only BMs

Patients with BMs and ECM had shorter IPFS than those with only BMs. The median IPFS was 6.8 versus 11.03 months (HR 0.573, 95% CI 0.399–0.821, *P* = 0.002). IPFS at 12 months was 28.6 versus 44.2%, and IPFS at 18 months was 8.2 versus 28.8% (Fig. 4).

#### Analysis of IORR, IDCR and DBF rates

Twelve individuals received immunotherapy before BMs, and the median time was 4.77 months (ranging from 1.6 to 14.47 months) after treatment with immunotherapy plus chemotherapy. When BMs were assessed, six patients stopped immunotherapy afterward.

On the day of radiotherapy initiation, the IORR rate was higher in Group B, but the IDCR rates were similar between Groups A and B. The IORR rates were 72.0% in Group A and 89.6% in Group B (*P* = 0.012). The IDCR rates were 93.6% and 93.8%, respectively (*P* = 0.976).

Forty-eight of 133 (36.1%) patients in Group A and 16 of 25 (64.0%) people in Group B experienced DBF. The DBF rate was much higher in Group B than in Group A (*P* = 0.001). Eighty-five individuals in Group A and 9 in Group B in experienced local progression. Twenty-two patients in Group A died of rapidly progression of extracranial diseases before efficacy evaluation of intracranial diseases.

The intracranial and extracranial reactions were not exactly the same. Among 64 individuals, 34 (53.1%) had a better intracranial reaction, including 7 patients with the same reaction and 23 with a higher extracranial efficacy.

## Discussion

The management of SCLC with BMs is limited and essentially relies on radiotherapy. WBRT is the standard treatment for these patients. Tyler P. Robin et al. (2018) suggested that radiosurgery alone is associated with favorable outcomes for SCLC patients afflicted with isolated BMs. Rusthoven et al. (2020) considered SRS to be another choice for BMs that developed from SCLC. Compared to WBRT, SRS did not decrease OS. However, whether WBRT or SRS is the most appropriate treatment modality for BMs needs to be further evaluated in the era of immunotherapy.

According to the results from IMPOWER133 and CASPIAN, atezolizumab and durvalumab, two PD-L1 monoclonal antibodies, both showed antitumor activity in ES-SCLC patients. QZ et al. (Zhou et al. 2020) reported a case who achieved complete remission of local lesions after receiving durvalumab monotherapy as a third-line treatment. PD-L1 inhibitors seem to be a new choice for SCLC patients. Tumor cells downregulate the immune response and promote immune tolerance by expressing PD-L1 and binding PD-1 expressed on T cells. Immune checkpoint inhibitors (ICIs) can block the interaction (Keir et al. 2008) and stimulate the normal activity of immune cells to achieve an antitumor effect. In theory, both PD-1 inhibitors and PD-L1 inhibitors have antitumor activities. Nevertheless, evidence that SCLCs obtain OS or PFS benefit from PD-l inhibition is lacking (Gadgeel et al. 2018; Owonikoko et al. 2021; Rudin et al. 2020; Spigel et al. 2021). Fortunately, a meta-analysis (Yu et al. 2021) showed that SCLC patients exhibited an improved OS from PD-1 inhibitors plus chemotherapy compared to chemotherapy. In this study, approximately 24 patients in Group B received PD-L1 monoclonal antibody during treatment, 36 people received PD-1 monoclonal antibody, and the other 4 individuals accepted 2 types of drugs successively. Our study did not determine which type of drug provided a greater benefit. Compared to SCLC patients with BMs who only received CRT, OS, IPFS and IORR were significantly improved among patients who received CRT plus at least four cycles of immunotherapy. The result was similar to that reported by Schapira et al. (2018), who found that the concurrent treatment of PD-1 inhibitors and radiotherapy improved the OS (median OS 17.6 months) and locoregional disease control of NSCLC patients with BMs. In addition, a phase two study of tislelizumab in combination with platinum-based treatment for ES-SCLC also showed promising results. The median PFS of SCLC patients was 6.9 months, and the median OS was 15.6 months (Wang et al. 2020). Furthermore, a case report using pembrolizumab alone as SCLC third-line treatment achieved a complete response (Zhang et al. 2020).

As demonstrated, radiotherapy could modulate the immunogenicity of tumor cells. Unfortunately, it rarely generates durable therapeutic responses. Repeated radiation treatments always lead to serious side effects. Prolonging therapeutic responses seems to be a good method to improve therapeutic effects. Dovedi et al. (2014) observed enhanced therapeutic efficacy from a combination of radiotherapy and immunotherapy. They found increased PD-L1 expression caused by low doses of local fractionated-dose radiotherapy delivered as 10 Gy in 5 fractions, which was consistent with the results of Dovedi et al. (Sharabi et al. 2015). Furthermore, many studies have shown that radiation can enhance the adaptive immune system. Anurag Gupta et al. (2012) reported that irradiation could activate tumor-associated dendritic cells, improving antigen presentation to T cells. Eric A. Reits et al. (2006) demonstrated that irradiation could upregulate the expression of MHC class I molecules to reinforce detection by the immune system. Simon J. reported that the enhanced immune system activity caused by radiotherapy may have aided in the improvement.

Unfortunately, we did not observe a significant improvement in IDCR in Group B. Prerna Guleria (2020) reported that PD-L1 expression in SCLC was extremely low with only approximately 3% of cells expressing PD-L1. Ryul Kim et al. (2019) suggested that the levels of PD-1 + TILs were significantly decreased in BMs compared with primary lung lesions. In this study, PD-L1 expression was detected in only six patients. In one patient, 30% of cells expressed PD-L1, whereas less than 3% expression was noted in the remaining five patients. This finding may explain why the survival benefit was not obvious among SCLC patients compared with NSCLC patients.

Our data indicated that both immunotherapy and ECM were related to the OS of SCLC patients with BMs. We found that many patients passed away from uncontrolled extracranial diseases rather than BMs, which was confirmed by Riihimaki et al. (2014). They suggested that SCLC patients were more likely to die of liver metastases or bone metastases rather than brain metastases. In their study, the 1-year survival rate was only 19% among patients with liver metastases, while the rate was approximately 41% among patients with brain metastases. Denise et al. (Bernhardt et al. 2018) suggested that ECM status significantly impacts the OS of SCLC patients with BMs, and those with controlled or stable ECM disease had a longer median OS than those with progressively or not controlled ECM. IMPOWER133 (Goldman et al. 2021) suggested that SCLC patients with liver metastases exhibited improved OS after treatment with a PD-L1 inhibitor plus chemotherapy. Thus, we hypothesized that the addition of immunotherapy would contribute to the management of extracranial diseases and subsequently the prolonged OS of Group B.

SCLC patients with BMs benefit from chemotherapy plus immunotherapy and radiotherapy for BMs. The timing of the immunotherapy is not clear. The results of KEYNOTE-189 (Gadgeel et al. 2020) indicated that applying pembrolizumab as a first-line treatment rather than second-line treatment resulted in longer PFS for NSCLC patients. However, PD-1 monoclonal antibody did not show antitumor efficacy as either first-line (Rudin et al. 2020) or second-line (Spigel et al. 2021) treatment. Moreover, some patients in Group B used ICIs across the lines, suggesting the role of continuous use of ICIs in prolonging the OS of SCLC patients.

We acknowledge the limitations of our retrospective analysis. This is a single-institution analysis. Few patients with poor ECOG status were enrolled in our study, which might result in an overestimation of the efficacy of immunotherapy. Patients in Group B were administered different types of immune inhibitors, which might influence the outcome. Approximately 14% of patients underwent brain CT scans for follow-up on brain metastases, and this modality might lead to missed diagnosis of small BMs. Furthermore, treatment-related brain necrosis, memory deterioration and cognitive disorders were not analyzed in this study.

Further prospective clinical studies of the treatment for extensive-stage SCLC patients should be performed.

## Conclusion

Immunotherapy improved the OS and intracranial IPFS of SCLC patients with brain metastases. Our study supported the notion that at least four cycles of ICIs should be applied for SCLC patients with BMs, and cross-line treatment with ICIs is recommended. Given the low survival rates of SCLC patients, we advise the use of ICIs as early as possible.

## Data Availability

Datasets generated and analyzed during the study are available from JYC on reasonable request.
